# Knowledge, perceptions and practices towards diabetes risk in sub-Saharan Africa: a mixed-methods scoping review

**DOI:** 10.1017/S1368980024000752

**Published:** 2024-03-27

**Authors:** Anthony Muchai Manyara, Elizabeth Mwaniki, Jason MR Gill, Cindy M Gray

**Affiliations:** 1 School of Health and Wellbeing, University of Glasgow, Glasgow, UK; 2 Department of Health Systems Management and Public Health, Technical University of Kenya, Nairobi, Kenya; 3 Global Health and Ageing Research Unit, Bristol Medical School, University of Bristol, Bristol, UK; 4 School of Cardiovascular and Metabolic Health, University of Glasgow, Glasgow, UK; 5 School of Social and Political Sciences, University of Glasgow, Glasgow, UK

**Keywords:** Diabetes, Africa, Knowledge, Perceptions, Practices

## Abstract

**Objective:**

To synthesise current evidence on knowledge, perceptions and practices towards type 2 diabetes risk in sub-Saharan Africa

**Design:**

Mixed-methods scoping review, which included 101 studies (seventy-three quantitative, twenty qualitative and eight mixed methods) from seven electronic databases.

**Setting:**

Sub-Saharan Africa, 2000–2023.

**Participants:**

Men and women without diabetes with mean ages ranging from 20 to 63 years.

**Results:**

The majority of participants in most studies knew the three main diabetes modifiable risk factors – excess weight, unhealthy diet and physical inactivity. However, most people with excess weight in almost all studies underestimated their weight. Further, the self-described ideal body weight was between midpoint of normal weight and the upper limits of overweight in most quantitative studies and was described as not too skinny but not too fat in qualitative studies. In the majority of studies, participants reported low engagement in weight control, high regular sugar intake, and low regular fruit and vegetable intake but moderate to high engagement in physical activity. Barriers to reducing diabetes risk were social (e.g. societal perceptions promoting weight gain) and environmental (e.g. limited affordability of healthy foods, high accessibility of Western diets and lack of physical activity facilities).

**Conclusion:**

There is a need for multicomponent type 2 diabetes prevention interventions that increase knowledge of identifying diabetes risk (e.g. what constitutes excess weight) and create social and physical environments that support healthy lifestyles (e.g. societal perceptions that promote healthy living, increased availability and affordability of healthy foods and physical activity facilities).

Diabetes is increasing rapidly in sub-Saharan Africa (SSA), imposing a significant health and economic burden^(1)^. Type 2 diabetes, accounting for most diabetes cases, is largely preventable through targeting lifestyle-related risk factors – excess weight, unhealthy diets and physical inactivity^(2)^. Current evidence suggests that lifestyle-related diabetes risk factors are becoming more prevalent in SSA. For example, pooled analyses from SSA surveys suggest that adiposity is on the rise: increase in BMI by a mean of 2 kg/m^2^ in men and 3 kg/m^2^ in women between 1980 and 2014^(3)^ and doubling of obesity between 1990s and 2014 in most countries^(4)^. Furthermore, pooled data from several SSA countries reported inadequate fruit and vegetable intake^(5)^ and overconsumption of carbohydrates, that is, carbohydrates as percentage of dietary energy supply^(6)^. Moreover, there is some evidence of a physical activity transition in SSA: for example, a systematic review of studies in school-aged children reported that urbanisation was associated with reduction in physical activity levels over time^(7)^. Therefore, there is an urgent need for lifestyle-based interventions to prevent and manage type 2 diabetes if SSA countries are to achieve the global target of reducing diabetes-related premature mortality by one-third by 2030^(8)^.

To inform diabetes preventive interventions and policies, there is a need to understand the drivers of unhealthy lifestyles. Such drivers are principally society-level factors (e.g. unavailability of healthy foods and sedentary occupations) but also individual-level factors (i.e. less healthy lifestyle choices and low risk awareness)^(9)^. There is emerging evidence on the factors contributing to unhealthy lifestyles in SSA, including some evidence syntheses. For example, a recent systematic review of quantitative studies (*n* 6) of diabetes knowledge in SSA schools reported poor knowledge of diabetes management and prevention among students and teachers^(10)^. A recent synthesis of qualitative studies (*n* 17) of SSA women and adolescent girls reported that sociocultural preferences for large body sizes and barriers to healthy eating and physical activity contributed to high obesity levels^(11)^. Another systematic review, including quantitative and qualitative studies (*n* 23), of determinants of dietary and physical activity behaviour in urban women of reproductive age, reported that knowledge and people’s social and physical environment among other factors influenced dietary and physical activity practices^(12)^. However, a comprehensive synthesis of knowledge and drivers of diabetes risk that extends beyond a particular setting (e.g. schools), gender (e.g. females) and methodological approach (e.g. quantitative or qualitative) is lacking. The current systematic scoping review therefore aims to fill this gap by synthesising current evidence on knowledge, perceptions and practices towards diabetes risk in SSA across different settings, genders and methodologies to inform primary diabetes prevention interventions.

## Methods

A systematic scoping review was considered more appropriate than a traditional systematic review to produce a broad and in-depth understanding of knowledge, perceptions and practices towards diabetes risk through inclusion of all relevant studies regardless of study design. The scoping review was guided by the methodological framework proposed by Arksey and O’Malley^(13)^, as described below.

### Step one: formulating a research question

The research question was developed by combining a broad question and a specific area of inquiry^(12)^. This included defining: the concepts as *knowledge about, perceptions and practices towards modifiable diabetes risks*; the health outcome of interest as *diabetes;* and the target population as *people in sub-Saharan Africa.* This process resulted in the overarching research question: what are the knowledge levels, perceptions and practices towards diabetes risk (i.e. weight, diet and physical activity) in SSA?

### Step two: identifying relevant studies

A systematic search of seven electronic databases, PubMed, Scopus, MEDLINE, Web of Science, Cumulative Index of Nursing and Allied Health Literature (CINAHL), African Journals Online, and PsycINFO, was conducted between December 2018 and February 2019, updated in March 2021 and October 2023. Search terms were based on three concepts from the review question: 1) ‘knowledge’, ‘perception’ and ‘practice’; 2) ‘diabetes’, ‘weight’, ‘diet’ and ‘physical activity’; and 3) ‘sub-Saharan Africa’. Keywords and indexing terms (MeSH and Emtree) and their synonyms were combined using the Boolean operators AND for concepts and OR for synonyms. Search terms were adapted for each database. Table 1 presents search terms used in PubMed, and the search strategy of all databases is in the Supplementary File. Reference lists of all included articles were hand-searched for relevant studies.

**Table 1:** Search terms used in PubMed database

Search	Query
#4	Search ((#1) AND #2) AND #3
#3	Search “Africa”[Mesh] OR “Africa South of the Sahara”[Mesh]OR (“Congo”[Mesh] OR “Tanzania”[Mesh] OR “Guinea-Bissau”[Mesh] OR “Eritrea”[Mesh] OR “Comoros”[Mesh] OR “Equatorial Guinea”[Mesh] OR “Zimbabwe”[Mesh] OR “Zambia”[Mesh] OR “Uganda”[Mesh] OR “Togo”[Mesh] OR “Swaziland”[Mesh] OR “Sudan”[Mesh] OR “South Africa”[Mesh] OR “Somalia”[Mesh] OR “Sierra Leone”[Mesh] OR “Senegal”[Mesh] OR “Rwanda”[Mesh] OR “Nigeria”[Mesh] OR “Niger”[Mesh] OR “Namibia”[Mesh] OR “Mozambique”[Mesh] OR “Mauritania”[Mesh] OR “Mali”[Mesh] OR “Malawi”[Mesh] OR “Madagascar”[Mesh] OR “Liberia”[Mesh] OR “Lesotho”[Mesh] OR “Kenya”[Mesh] OR “Cote d’Ivoire”[Mesh] OR “Ghana”[Mesh] OR “Gambia”[Mesh] OR “Gabon”[Mesh] OR “Ethiopia”[Mesh] OR “Chad”[Mesh] OR “Central African Republic”[Mesh] OR “Cameroon”[Mesh] OR “Burundi”[Mesh] OR “Burkina Faso”[Mesh] OR “Botswana”[Mesh] OR “Benin”[Mesh] OR “Angola”[Mesh] OR “Sao Tome and Principe”[Mesh] OR “Democratic Republic of the Congo”[Mesh]
#2	Search ((((((((“Diabetes Mellitus”[Mesh]) OR (“Body Weight”[Mesh] OR “Ideal Body Weight”[Mesh] OR “Weight Loss”[Mesh] OR “Weight Gain”[Mesh])) OR “Body Size”[Majr]) OR “Overweight”[Mesh]) OR “Obesity”[Mesh]) OR (“Diet”[Mesh] OR “Diet, Western”[Mesh] OR “Diet, Mediterranean”[Mesh] OR “Healthy Diet”[Mesh])) OR (“Food”[Mesh] OR “Food Quality”[Mesh])) OR “Exercise”[Mesh]) OR “Sedentary Behavior”[Mesh]
#1	Search ((((((“Knowledge”[Mesh] OR “Health Knowledge, Attitudes, Practice”[Mesh]) OR “Attitude to Health”[Mesh]) OR “Awareness”[Mesh]) OR “Perception”[Mesh]) OR “Culture”[Mesh]) OR “Comprehension”[Mesh]) OR “Health Behavior”[Mesh]

### Step three: study selection

The search results were exported to Endnote for the removal of duplicates and articles not in English. Titles and abstracts of the remaining articles were screened for eligibility before full texts were read to identify articles that met the inclusion criteria as follows: first, articles had to report peer-reviewed empirical studies published between 2000 and 2023. The year 2000 was chosen as the start date, as it was at the start of the third millennium when non-communicable diseases were identified as increasing in prevalence in low- and middle-income countries such as those in SSA^(14)^. Second, studies had to have been conducted in SSA countries (in part or entirely) with SSA defined as the region below North Africa consisting of forty-eight countries^(15)^. Third, the study population had to be adults (aged 18+ years), as type 2 diabetes is more prevalent in adults^(16)^. Fourth, the study sample had to be broadly healthy, defined as over 50 % of participants being free of diabetes or CVD. This criterion reflected the likelihood that patients may be exposed to information about lifestyle risk factors during contact with the health system. Finally, articles were included if they reported factors relevant to the research question, for example, knowledge levels of diabetes risk factors and perceptions of weight.

### Step four: charting the results

Data were extracted (by AMM) using a tool with the following sections: author and publication year, country, study aim, study design and methods, study setting and sample size, and findings relevant to the review question.

### Step five: synthesis and reporting the results

Quantitative data were synthesised narratively and presented in proportions, and some descriptive data (e.g. distribution of studies by country) were presented in graphs. Qualitative results were exported to NVivo 12 and thematically synthesised by: coding results (including quotes from study participants) line by line and combining codes into themes^(17)^. Quantitative sections of mixed-methods studies were synthesised with quantitative studies, while qualitative sections were synthesised with qualitative studies. Quantitative and qualitative data were combined using either a complementary approach (studies adding to each other) or confirmation/ refutation approach (studies supporting or contradicting each other)^(18,19)^. The quantitative and qualitative findings are reported under three main headings: weight, diet and physical activity. To illustrate qualitative findings particularly those related to perceptions, quotes from studies conducted in various countries were selected and are presented verbatim.

## Results

### Summary of search results

**Fig. 1:**
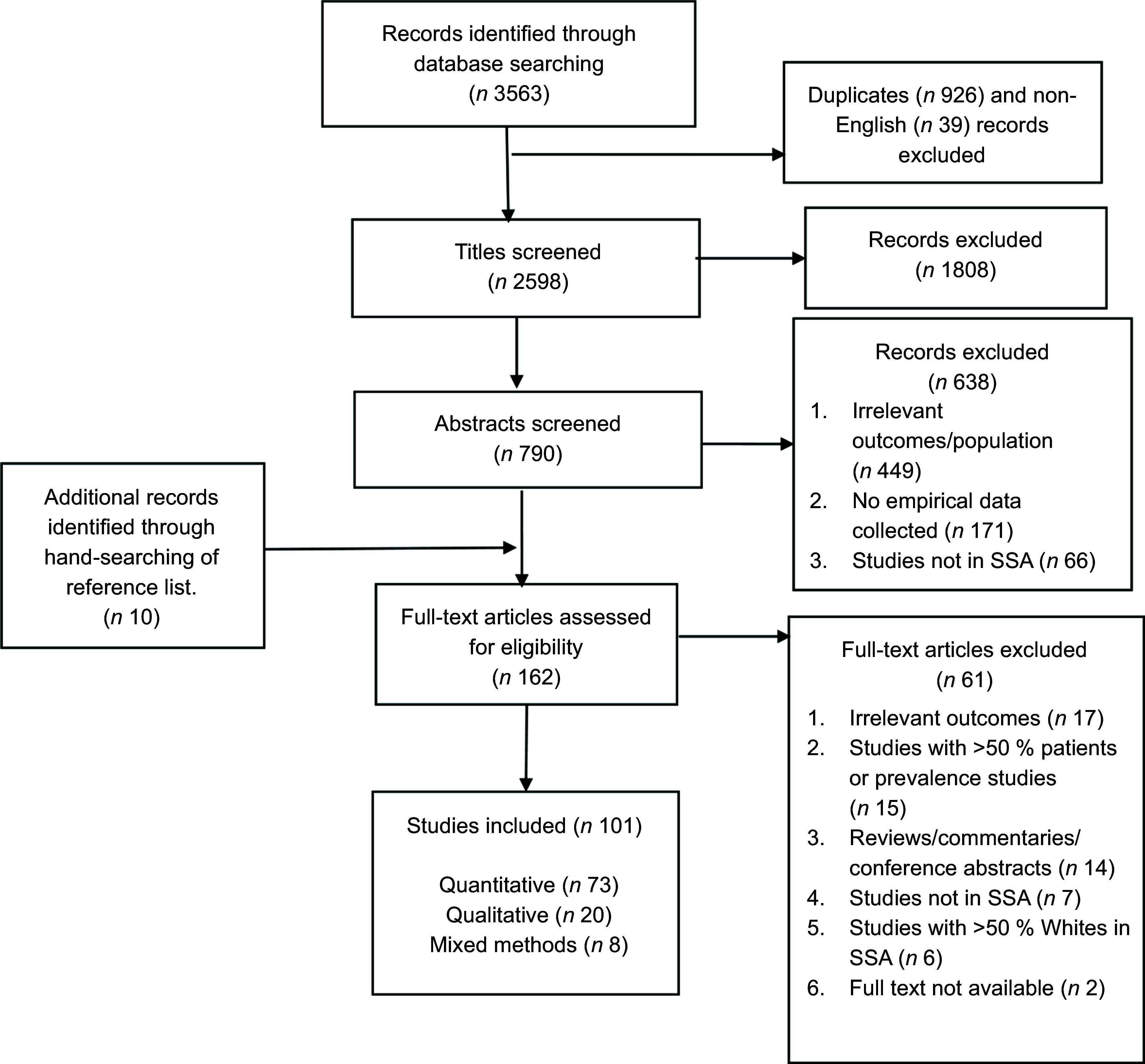
Literature search flow diagram. SSA, sub-Saharan Africa

Figure 1 shows the literature search flow diagram. A total of 3563 records were identified from the electronic search; 965 were excluded as they were either duplicates or not in English. A total of 1808 and 638 records were excluded after titles and abstracts screening, respectively. The full texts of the remaining 152 records and a further ten records identified through hand-searching were assessed. Fifty-nine full texts did not meet the inclusion criteria and were excluded at this stage; a further two full-text articles could not be accessed through the inter-library loan service at the University of Glasgow and were also excluded. Finally, 101 studies were included in the narrative synthesis. See a list of all included studies in the see online supplementary material, Supplementary File (Table 1).

### Study characteristics

**Fig. 2:**
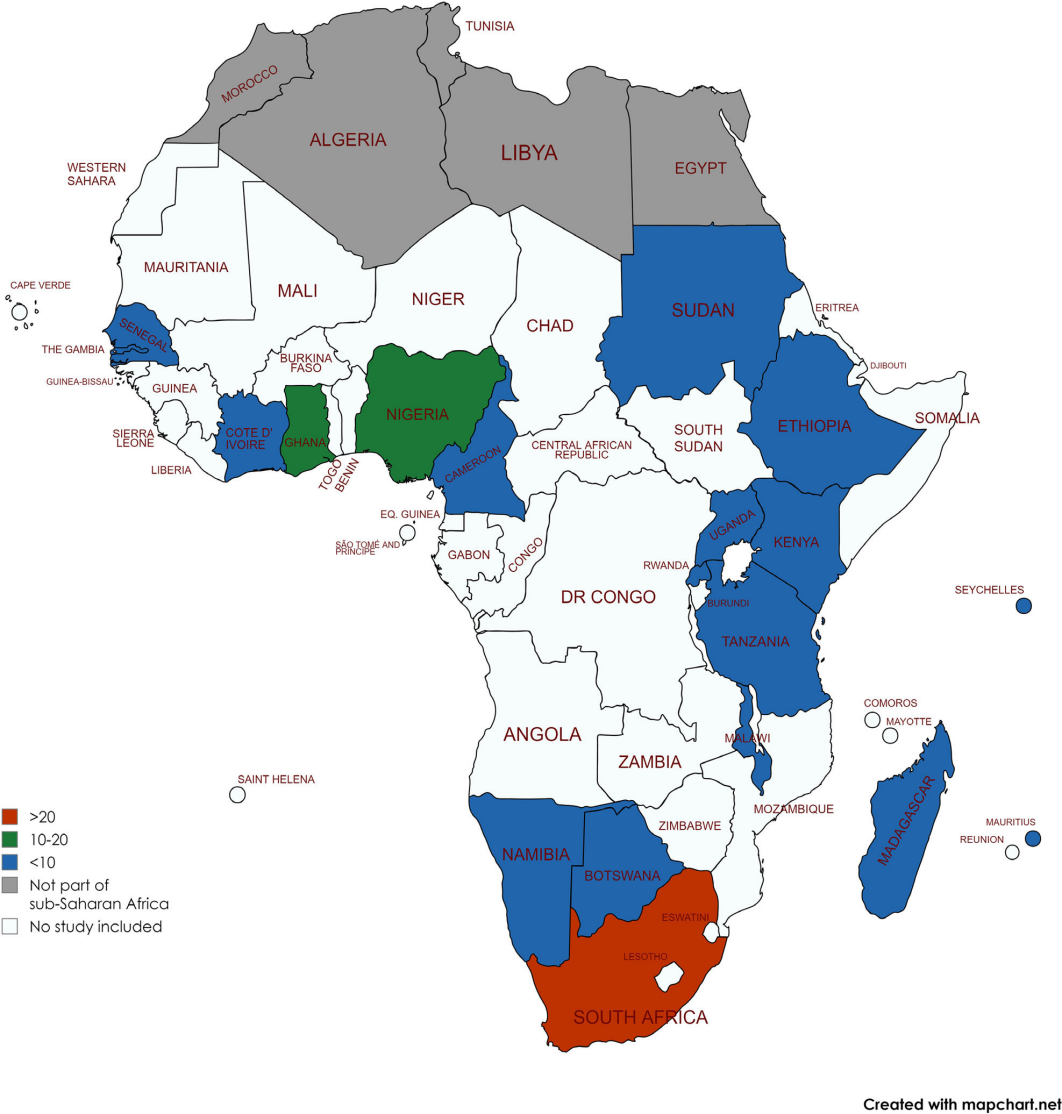
Distribution of studies by SSA country. SSA, sub-Saharan Africa

Figure 2 presents the distribution of studies across the nineteen SSA countries represented. The majority of studies were conducted in South Africa (*n* 40, 40 %), Nigeria (*n* 16, 16 %) and Ghana (*n* 13, 13 %). Most studies (74/101, 73 %) were conducted between 2011 and 2023, using quantitative methods (73/101, 72 %); twenty (20 %) were qualitative and eight (8 %) used mixed methods. Quantitative data were collected using a combination of questionnaires (81/81, 100 %), anthropometric measurements (40/81, 49 %) and body size silhouette show cards^(20)^ (20/81, 25 %). Qualitative data collection used in-depth interviews (17/28, 61 %) and focus group discussions (18/28, 64 %). The majority of studies (*n* 60, 59 %) were conducted in urban-only settings and the rest in: universities (*n* 19, 19 %); both rural and urban settings (*n* 15, 15 %); and rural-only settings (*n* 8, 8 %). The sample sizes ranged from 43 to 6628 for quantitative studies, with a third of the studies having a sample of ≥500, and 16–163 for qualitative studies, with about a half of the studies having a sample of ≥50.

### Weight

#### Knowledge of weight as a diabetes risk factor

Sixteen quantitative, six qualitative and one mixed-methods study explored whether participants knew that excess weight was a diabetes risk factor. In most quantitative studies, knowledge of risk associated with excess weight was moderate to high. In 10/16 (62 %) of the quantitative studies, the majority of participants (56–95 % of participants) mentioned excess weight as a risk factor^(21–30)^, while in the remaining six studies (38 %), 5–44 % did so^(31–36)^. In the qualitative studies, participants were aware of the health consequences of excess weight, including diabetes, in South Africa^(24,37,38)^ and Ghana^(39)^. Nevertheless, in an in-depth interview in Cameroon, hardly any participants associated excess weight with diabetes although they acknowledged it was a hypertension risk factor^(40)^. Furthermore, qualitative studies in South Africa found that people did not perceive the possible health risks of having excess weight^(41,42)^.

#### Perceived current and ideal weight

Widespread knowledge of excess weight as a diabetes risk factor did not translate to positive perceptions of normal weight: the majority of people underestimated their weight and chose an overweight body weight category as the ideal body weight. In total, thirty-one quantitative, six qualitative and four mixed-methods studies explored perceptions of current or ideal weight. Twenty-one quantitative studies investigated participants’ perceptions of their current body weight. The proportion of overweight/obese people who underestimated their weight ranged from 35 % to 98 % and was >50 % in almost all studies (19/21, 91 %)^(29,43–62)^. A South African qualitative study suggested that this underestimation may be due to normalisation of excess weight (i.e. being overweight perceived as normal) and a misconception that obesity referred to morbid obesity^(63)^

Sixteen quantitative or mixed-methods studies used a body size scale to investigate perceived ideal weight. Studies reported perceived ideal weight either as a mean of the body scale (*n* 10) or the percentage of participants who chose different clinical body weight categories (i.e. normal, overweight and obese) as their ideal body weight (*n* 6). Generally, in studies reporting the mean, perceived ideal weight was between the midpoint of clinical normal weight and the upper limits of overweight in both women and men in rural and urban settings^(61,64–72)^. In studies that used percentages, most young people preferred a normal-weight silhouette, while the majority of middle-aged adults preferred an overweight or obese body weight. Specifically, in two university studies, the majority of male students (56 %) in Nigeria^(57)^ and almost all female students in Nigeria and South Africa (≥90 %) preferred a normal weight as their ideal body weight^(57,73)^. In five community studies, the majority of men (53 % and 83 %) in Kenya and Ghana^(56,74)^ and women (51–74 %) in Ghana and South Africa chose an overweight or obese body size^(74–77)^ as their ideal body weight.

Box 1 shows qualitative findings on perceptions of the ideal body weight drawn from eight studies (qualitative (*n* 6), mixed methods (*n* 2)). Generally, the perceived ideal weight appeared to be overweight which was described in various ways. Furthermore, a person’s current body shape, which had various descriptions, influenced the acceptability of high body weight.

Box 1 Description of the perceived ideal weight and influences of high body weight acceptability

**The perceived ideal weight appeared to be overweight, described as:**

- ‘*not too skinny but not too fat’* in Ghana^(79)^
- ‘*not too thin or too fat’* in South Africa^(95)^
- *‘physically not too fat or small’* in Cameroon^(40)^.
- A South African community health worker pointed at a moderately overweight woman (BMI 27 kg/m^2^) and recounted: *‘This is what we mean by a proper woman, this woman is full, everyone sees that she is healthy and can do whatever work is required of her’* ^(24)^.

**Body shape influenced the acceptability of high body weights:**

- Even weight distribution – referred to as *‘stoutness’* in Cameroon^(71)^, *‘well-built but not like obese’* ^(136)^ *or ‘fuller figure not fat women’* ^(92)^ in South Africa, ‘*proportional body frame’* ^(39)^, ‘*Coca-Cola shape’* or having ‘*some hips’ and ‘a flat stomach’* in Ghana^(79)^ – was acceptable
- Uneven weight distribution –referred to as ‘*fatness’* in Cameroon^(71)^ or *‘tummy being too big’* in Ghana^(79)^ – was unacceptable

#### Societal perceptions influencing weight control

A total of twenty-two studies explored societal perceptions related to weight: eight quantitative, twelve qualitative and two mixed methods. Generally, these studies found that societal perceptions may positively or negatively influence weight control. Being overweight/obese was associated with positive attributes such as health, respect, likeability, affluence, attractiveness and maturity in five quantitative studies in Nigeria, Ghana and South Africa^(24,70,73,76,78)^. On the other hand, normal weight was associated with positive attributes such as confidence, femininity (in women), happiness, strength, respect, and high willpower in two quantitative studies in South Africa and Senegal^(72,73)^. Obesity was associated with negative attributes such as greed, unattractiveness, social undesirability, lack of respect, unhappiness, clumsiness and not being a potential spouse in seven quantitative studies in Ghana, Nigeria, Senegal and South Africa^(24,45,51,70,72,73,76)^. A Nigerian study found that positive and neutral perception of large body weight was associated with higher chances of currently being obese^(62)^. Furthermore, South African and Ghanaian qualitative studies reported that excess weight negatively affected social relationships, and for some, it was viewed as being handicapped, something to be ashamed of and stigmatised^(37,39,79)^

Box 2 summarises societal perceptions that may promote weight gain or weight control from the qualitative studies. The main perceptions that influenced weight gain included associating high body weight with financial stability, respect, attractiveness (mainly in women) and good health; while the decline in social acceptability of high body weight due to health implications promoted weight control.

Box 2 Societal perceptions that may promote weight gain and weight control in qualitative studies

**Societal perceptions that may promote weight gain.**

- A high body weight was associated with: financial stability *‘a sign of good living’* in urban Ghana^(39)^, urban Cameroon^(40)^ and urban South Africa^(38,63)^; being *‘well-off’* in rural and urban Uganda^(91)^; and ‘*having a lot of money’* in urban South Africa^(41)^ and urban Cameroon^(64)^.
- A high body weight attracted respect: *‘administrative belly’, ‘executive belly’, ‘commanding belly’* in Cameroon^(40,64)^, and *‘a very important person’* in Uganda^(91)^.
- A high body weight in women was associated with good care from husbands in Malawi^(100)^, Cameroon^(40,64)^ and South Africa^(95,109,136)^. For example, a Cameroonian woman recounted: ‘*For a woman, being overweight suggests that her husband takes good care of her’* ^(64)^.
- A high body weight was considered attractive in women: *‘true buttocks’* in Cameroon^(40)^; *‘revealing the female figure’* ^(37)^, *‘big hips’* ^(38)^ and *‘fresh and round’* ^(24)^ in South Africa; *‘presentable’* in Ghana^(39)^; *‘nice and beautiful’* in Malawi^(100)^; *‘beautiful and attractive’* in South Africa^(63)^.
- High body weight was viewed as part of African culture in South Africa^(37,41,111)^ and Ghana^(79)^. For example, an obese South African woman mentioned that: *‘According to our values and culture, it is important for a woman to have a large body’* ^(41)^.
- Nudges by spouses, family and peers to gain weight in Ghana^(39,79)^: *‘my husband wants a fat person so he wants me to gain some more weight’* ^(39)^.
- Association of weight loss with ill health, especially HIV/AIDS in South Africa^(24,38,63,92,111,135)^ and Uganda^(91)^. A South African woman recounted: ‘*People are actually scared of like losing weight because they will say that they are sick, people will say you have HIV’* ^(135)^.
- Association of weight loss with financial constraints in Uganda^(91)^ and South Africa^(37)^. A Ugandan obese man said *‘If you lose weight people think you no longer have money’* ^(91)^.

**Societal perceptions that may promote weight control**

- Decline in acceptability of high body weight due to its health implications in Cameroon^(40)^, Ghana^(39,79)^ and South Africa^(37,41,92,136)^. A South African woman said: ‘*A woman these days for her health’s sake should not be overweight’* ^(41)^.
- Low body weight was no longer associated with HIV/AIDS in South Africa: A participant recounted: *‘it used to be like that [association of weight loss with HIV] but due to the presence of ARVs (anti-retroviral), it’s not a problem anymore; you will see a person fat with her ARVS’* ^(136)^.
- Low body weight was perceived as attractive in Cameroon^(40)^ and South Africa^(37,38)^. South African authors reported that: *When shown the picture of a slender woman, most of the participants agreed that she was ‘nice looking’, healthy and sexy* ^(37)^.
- High body weight was not specifically perceived as a sign of financial stability in Uganda^(91)^ and South Africa^(37,92)^. A South African participant felt: *‘even if you have money, you can be fat, even if you don’t have money you can be fat’* ^(37)^.

#### Weight control

Given the widespread weight underestimation, idealisation of overweight and societal perceptions undermining weight control, it was not surprising that weight control practices were not commonly reported in the sixteen quantitative and one mixed-methods study exploring weight control practices. Although a majority of participants (>50 %) in four studies in Ghana, Seychelles and South Africa had attempted weight control^(53,80–82)^, in most studies (*n* 13/17, 76 %), only 6–40 % of participants reported ever trying to control their weight^(23,29,44,46,49,50,58,62,71,83–86)^.

A subset of studies (*n* 8) explored weight control methods used. The most commonly used methods were reducing energy intake (6–62 % of participants in seven studies^(29,44,49,71,83,85,86)^) and increasing physical activity (13–58 % of participants in four studies^(29,49,83,85)^). Other weight control methods mentioned were slimming tablets (3–9 % of participants in four studies^(44,49,83,85)^), taking hot water (<25 % of participants in two studies^(24,85)^), and taking herbal tea and lemon juice (17 % and 9 % of participants, respectively, in one study^(24)^). Similarly, in the four qualitative studies that explored weight control, the main practices reported were dietary changes, such as reducing foods high in sugar and fat^(37,41)^ and starchy foods^(38,39)^, and increasing physical activity^(37–39,41)^. Other reported weight control strategies included taking Chinese herbal medicines^(39)^ and slimming tablets^(37,41)^, using a weight loss belt^(37)^, modifying meal times^(39)^ and smoking^(41)^.

### Diet

#### Knowledge of unhealthy diet as a diabetes risk factor

Twelve quantitative, five qualitative and one mixed-methods studies explored diet as a diabetes risk factor. In all quantitative studies, 47–98 % of participants mentioned unhealthy diets, particularly high sugar intake, as a diabetes risk factor^(21,22,26,27,30,32–34,36,87–89)^. This was consistent with the qualitative findings, which reported that high sugar intake was the main perceived cause of diabetes, probably due to diabetes being referred to as the ‘sugar disease’ in local languages in SSA^(22,40,90,91)^. However, knowledge gaps were identified: particularly the role of excess energy in diabetes development was less well understood. For example, some focus group participants in South Africa thought that it was fat rather than sugar which led to diabetes risk from excess weight^(92)^, and a qualitative survey in Cameroon found that some participants thought diabetes risk would be reduced by taking bitter drinks (such as alcohol) instead of sugary soft drinks^(40)^.

In relation to fruit and vegetable intake being protective of diabetes, only 20 % of participants in a quantitative study in urban Senegal^(93)^ knew of the protective role of fruit and vegetable intake, in contrast to 73 % in urban Nigeria^(36)^. Additionally, a qualitative study with women in urban Uganda found a lack of understanding of the protective role of fruit and vegetables^(94)^.

#### Perceived barriers and facilitators of healthy eating

Thirteen qualitative studies explored perceived barriers and facilitators to healthy eating. The most commonly reported barrier was limited accessibility to healthy foods. This was due to the: (1) high cost of healthy foods, such as fruits and vegetables, in South Africa^(22,37,38,63,94–97)^, Uganda^(94,96)^, Nigeria^(97)^, Cameroon^(40)^ and Ethiopia^(98)^; and (2) limited availability of healthy foods due to seasonality of fruits and vegetables in Cameroon^(40)^ and Uganda^(94,96)^, limited local production in Ethiopia^(98)^ and Uganda^(91,99)^, and limited healthy food options in a South African^(38)^, Malawian^(100)^, and Ugandan^(96)^ workplaces or restaurants. Additionally, the availability and desirability of Western diets (i.e. their association with high socio-economic status) was widely reported as a barrier to healthy eating in South Africa^(22,38,63,95,99,101)^ and Uganda^(94)^. Traditional dietary practices were another commonly cited barrier, and these included consumption of starchy staple foods in Cameroon^(40)^ and South Africa^(37)^, excessive use of palm oil in cooking in Cameroon^(64)^, salty and oily food in Uganda^(96)^, and high sugar intake with coffee in Ethiopia^(98)^. Finally, unhealthy diets were perceived as tasty, and giving them up was seen as ‘*sacrificing a good life’* in Uganda^(91)^ and Ethiopia^(98)^. Proposed facilitators of healthy eating were a gradual change from unhealthy to healthy diets in Uganda^(91)^, and preparing healthy foods such as vegetables in an ‘*exciting, tasty way’* in South Africa^(95)^. Also, social support, such as in the family or workplace, had facilitated healthy eating in South Africa^(38)^, Uganda^(96)^ and Nigeria^(97)^. Furthermore, knowledge and perception of the benefits of healthy eating was reported as a facilitator of healthy eating in Uganda^(94,96)^ and Nigeria^(97)^.

#### Dietary practices

Eight quantitative studies and three qualitative studies explored dietary practices: specifically, sugar intake, and fruit and vegetable consumption. Sugar intake was generally high: 55–78 % of participants regularly consumed foods or drinks high in sugar in South Africa^(102–104)^ and Nigeria^(89)^. Similarly, a qualitative study in Ethiopia reported high sugar intake, especially in coffee^(98)^. A qualitative study from South Africa reported that participants attributed regular consumption of sugary drinks to advertising, the accessibility of sugary drinks, habit and addiction^(101)^. However, women in urban Uganda reported that they limited sugar and oil during food preparation to prevent diabetes^(94)^.

Consumption of fruit and vegetables was very low in South Africa and Nigeria: less than a quarter of participants in two quantitative studies met the recommended daily fruits and vegetable intake^(34,105)^; general daily fruit or vegetable intake ranged between 10 and 34 % in three studies^(86,89,106)^; and only a quarter of health workers reported frequently consuming fruit and vegetables in one study^(103)^. Further, qualitative evidence from Ethiopia revealed that vegetables were not eaten daily, and fruit intake was low^(98)^.

### Physical activity

#### Knowledge of physical inactivity as diabetes risk factor

Nine quantitative studies explored physical inactivity as a diabetes risk factor, and in most studies, knowledge levels were moderate to high: 56–90 % of participants in 56 % of the studies (*n* 5)^(21,23,26,27,30)^ and 20–46 % in a 44 % of the studies (*n* 4)^(31,32,34,36)^ knew that physical inactivity was a risk factor. However, participants in a qualitative study in South Africa felt that people who were overweight were at risk of lifestyle diseases including diabetes because they did not exercise^(92)^. In contrast, the a qualitative study from Cameroon found that very few participants associated physical inactivity with diabetes^(40)^, and in Uganda the link between physical inactivity and disease, and the recommended physical activity levels were not understood^(94)^.

#### Perceived barriers and facilitators of engaging in physical activity

Box 3 presents the perceived motivators, benefits and barriers to engaging in physical activity reported in eleven quantitative studies. The most common motivators were peer and family support, and important perceived benefits included weight loss, better health, psychological benefits (e.g. increased self-esteem) and physical attractiveness. The main barriers to engaging in physical activity were limited availability and affordability of facilities, and perceived time constraints.

Box 3 Motivators, perceived benefits and barrier to engaging in physical activity in quantitative studies

**Motivators**

- Social support (e.g. company, peer and family support, encouragement)^(113,117,153,154)^
- Health concerns or weight loss^(107,153)^
- Active travel (e.g. walking and cycling) infrastructure and safety^(118,153)^
- Safe and accessible exercise facilities and infrastructure^(110,113,153,154)^
- Previous engagement in physical activity^(153,155)^

**Perceived benefits**

- Weight loss and better health^(113,117,119,153,155–157)^
- Physical attractiveness and psychological benefits (e.g. improved mental health, increased self-esteem, relaxation and stress reduction)^(113,117,119,153,155–157)^
- Improved physical performance (fitness, strength, stamina and endurance)^(117,119,155–157)^
- Opportunities for social interaction^(117,119,153,156)^

**Perceived barriers**

- Limited availability and affordability of exercise facilities/equipment^(107,110,120,153–156)^
- Perceived time constraints^(107,110,113,119,120,153,155)^
- Negative attitudes or low awareness of the importance of exercise^(110,153,155)^
- Low motivation^(120,154,155)^
- Exercise perceived as tiring^(119,120)^.

Qualitative findings on the perceived barriers and facilitators of physical activity were reported in thirteen qualitative and three mixed-methods studies. First, time constraints emerged as an important barrier in Cameroon^(40)^, Uganda^(94,96)^, Ghana^(39,107,108)^ and South Africa^(38,95,109)^. Second, lack of physical activity facilities or equipment was mentioned as a barrier in Ghana^(108)^, Uganda^(96)^ and South Africa^(37,38,41,63,95,110)^. Third, exercise as a form of physical activity was perceived as tiring in Uganda^(94)^ and Ghana^(107,108)^, and South Africa^(38,95)^. Fourth, some sociocultural perceptions contributed to the social undesirability of physical activity. These included association of exercise with the young in Ghana^(107)^, Uganda^(96)^, and South Africa^(37,111)^, tight sports attire being socially unacceptable for women in Uganda^(94)^ and South Africa^(24,111)^, traditional gender roles that discouraged girls from taking up sports in Uganda^(94)^ and South Africa^(95,111)^, and association of exercise with undesirable weight loss in South Africa^(24,111)^. Finally, safety concerns (specifically around high crime rates) were identified as a barrier to outdoor physical activity in Uganda^(94)^ and South Africa^(41,63)^. In relation to facilitators, fitting physical activity in daily schedules through active travel, household chores or simple leisure activities such as walking were seen as supporting physical activity in South Africa^(24,37,38,95)^ and Uganda^(91,96)^. Furthermore, exercising together in groups was mentioned as a facilitator in Uganda^(94,96)^.

#### Physical activity practices

Eight quantitative^(24,35,36,85,103,112–114)^, two qualitative^(41,42)^ and three mixed-methods^(108,111,115)^ studies explored physical activity practices. In the majority of quantitative studies (7/10, 70 %), 46–87 % participants regularly engaged in physical activity. In urban Ethiopia, 61 % of participants reported accumulating 30–60 min of physical activity ‘frequently or very frequently’^(23)^ and 76 % reported engaging in physical activity ‘sometimes or often’ in urban Ghana^(107)^. WHO physical activity recommendations were met by 46 % in urban Botswana^(116)^, 50 % in rural Nigeria^(34)^ and 86 % in urban South Africa^(110,112)^ (but only by 48 % of staff in a private hospital in South Africa^(113)^). However, 62 % of urban residents in Ghana^(35)^, 72 % of participants in four Kenyan regions and 80 % of South African taxi drivers did not report any regular exercise^(84,102)^.

Qualitative findings on physical activity engagement were varied. For instance, focus group participants in urban South Africa reported that they did not engage in vigorous physical activity^(41)^. In a mixed-methods study in Cameroon, participants from a rural setting felt that farming enabled them to engage in intense physical activity^(114)^. In another Cameroonian study that used in-depth interviews, most urban residents did not engage in exercise, but this was reported to be changing with more people engaging in leisure-time physical activity^(40)^.

### Factors associated with knowledge, perceptions and practices

Thirty-seven studies reported on factors associated with knowledge, perceptions and practices. Table 2 provides a summary of these factors for weight, diet and physical activity. Generally, there were gender differences in practices (weight control and dietary practices) and weight perceptions in the nineteen studies which disaggregated results by gender. Also, younger age and higher education were associated with positive perceptions towards healthy weight and weight control, while living in urban settings was associated with preference for a lower body weight, but also with unhealthy dietary practices. Furthermore, it was not conclusive if knowledge and perception of risk was associated with healthier weight and dietary of physical activity practices.

**Table 2:** Factors associated with weight, dietary and physical activity knowledge, perceptions, and practices

Factor	Factors associated (√) or not associated with weight, dietary and physical activity knowledge, perceptions, and practices		
	Knowledge	Perceptions	Practices
Gender	**X:** Differences in knowledge levels between men and women were not observed in two studies that included both sexes^(29,106)^.	**√:** Men had a higher weight underestimation in three studies^(34,67,77)^, while women had a higher underestimation in two studies^(29,75)^. **√:** Two urban studies in Senegal^(70)^ and Gambia^(68)^ found that women chose a higher ideal body weight than men. **√:** Three university studies found gender differences in perceived physical activity motivators, benefits and barriers^(117–120)^. X: In a Ghanaian study among civil students who had high knowledge levels, there were no significant gender differences in knowledge^(29)^.	**√:** Numerically more women than men had attempted weight control in Ghana^(121)^ (33 % *v*. 23 %) and Seychelles^(53)^ (62 % *v*. 45 %). **√:** In a Ghanaian study, more men than women (63 % *v*. 32 %) used exercise for weight loss, while more women than men modified their diet (67 % *v*. 37 %)^(29)^. **√:** Two studies found that daily intake of fruit was higher in women than men in South Africa: 34 % *v*. 24 %^(107)^ in one study and a positive correlation in favour of female gender in another study^(104)^. **√:** Women had better dietary practices than men in two South African studies^(106,122)^.
Social economic status	Higher income associated with better knowledge on diabetes risk factors^(31)^.	**√:** Weight underestimation was higher in individuals of low socio-economic status in Seychelles^(53,123)^. **√:** Preference for low body weight being reported in low and middle wealth category in comparison with lowest category in Ghana^(83)^. **√:** In Seychelles, men with a higher social economic status (SES) had a higher perceived ideal body weight than men of lower SES, and in contrast women with a higher SES preferred a lower ideal body weight compared to their peers with a lower SES^(68)^.	X:Socio-economic status was not associated with healthy dietary practices among South African university students^(106)^.
Age		**√:** Preference for a low body weight associated with younger age in Ghana^(71,124)^. **√:** In Ghana, being 40 years and older was associated with perceiving a larger ideal body size^(70)^. **√:** Weight underestimation was lower in overweight or obese people who were aged 50 years and older in Kenya^(125)^.	**√:** Older age was associated with healthy dietary practices in two South African studies^(86,126)^. X: Age was not associated with dietary practices among South African students^(106)^.
Education	Higher education level was associated with better knowledge on diabetes risk factors in studies that looked at various risk factors^(31,127–130)^. Women with tertiary level of education had more knowledge on weight being a diabetes risk factor awareness compared to those with secondary level of education in Nigeria^(78)^.	**√:** Preference for a low body weight associated with having a university-level education in Ghana^(71)^. **√:** In Nigeria, lower educational level was associated with viewing obesity as socially desirable^(131)^. **√:** In Ghana, a low educational level was associated with perceiving a larger ideal body size^(70)^.	
Residence – rural/urban		**√:** Preference of a lower ideal body weight was associated with living in an urban setting in first 12 years of life in Ghana^(83)^, residing in an urban compared to rural setting in South Africa^(86)^.	**√:** Residing in an urban setting for 15 years associated with higher intake of westernised diets and lower intake of traditional foods in Cameroon^(64)^. **√:** Rural residents ate more fruit and vegetables daily than semi-urban residents in South Africa^(132)^ **√:** In contrast to another South African study, semi-urban residents had better dietary practices including eating more fibre than rural residents^(86)^.
Knowledge on risk factor			**√:** A Nigerian study in a rural community found that having excess weight was associated with lack of knowledge on excess weight being a diabetes risk factor^(34)^. Two Nigerian studies found conflicting results on whether knowledge was associated with healthy eating: X: knowledge was not associated with healthy dietary practices in a rural setting^(34)^; **√:** knowledge was associated with healthy dietary practices among male postgraduate students^(89)^. X: In rural Nigeria, knowledge of physical inactivity as a diabetes risk factor was not associated with being physically active^(34)^. **√:** In contrast, a study among hospital staff in South Africa found that knowledge was associated with better physical activity attitudes and practices^(113)^. **√:** A South African study among obese participants found that 93 % had thought of losing weight, but 73 % had attempted weight loss with 94 % reporting lack or insufficient weight loss knowledge and skills^(82)^.
Perceptions			**√:** A Nigerian study found that perception of being obese was associated with likelihood to attempt weight loss^(62)^. X: In contrast, a Malawian qualitative study among urban women found those with a normal weight were involved in weight control, while those who were overweight or obese were not controlling their weight^(100)^. **√:** Discontent and concerns about body appearance (weight and size) were associated with lower physical activity engagement in Botswana^(116)^.

## Discussion

This review has presented a comprehensive synthesis of current evidence on practices, knowledge and perceptions in relation to lifestyle diabetes risk factors in SSA. Most people did not engage in weight control and had a low intake of fruit and vegetables and a high intake of sugar/sugary foods. However, most reported undertaking regular physical activity. The majority of participants in most studies were aware of the three main modifiable risk factors – excess weight, unhealthy diet and physical inactivity. However, there appeared to be a limited understanding of what constitutes a healthy weight, as most people with excess weight in almost all studies underestimated their weight. Furthermore, it was clear that some societal perceptions promoted weight gain or discouraged weight loss through the association of high body weight with financial stability, respect, attractiveness (mainly in women) and good health. However, such societal perceptions may be on the decline and being replaced by perceptions that promote weight control, such as increased understanding of the negative health implications associated with high body weights. The perceived barriers to consuming healthy diets included limited availability and affordability of healthy foods and the availability and desirability of Western diets. Similarly, the main perceived barriers to physical activity were the limited availability and affordability of physical activity facilities, as well as time constraints. Finally, this review found that younger age and higher education were associated with positive perceptions towards healthy weight, and residing in urban settings was associated with a preference for lower body weight, but unhealthy dietary practices. However, evidence on the associations between gender, knowledge and perception of risk, and healthier weight, dietary and physical activity practices was inconclusive.

Despite moderate to high knowledge of weight as a diabetes factor in SSA, more detailed knowledge appears to be lacking (such as what constitutes a healthy weight), which may have contributed to the majority of overweight/obese participants underestimating their weight. Our finding that many SSA men underestimate their weight is consistent with evidence from high-income countries, where 48–55 % of men underestimated their weight but contrasts findings in women from high-income countries where only 23–31 % underestimated their weight^(115)^. The high levels of weight underestimation apparent in the current review may have contributed to the low levels of engagement in weight control reported in most of the included studies. Systematic review evidence from high-income countries suggests that perceiving oneself as overweight is associated with a higher likelihood of attempting weight loss^(133)^. However, perceptions of being overweight have also been associated with unintended consequences, such as unhealthy weight control, stigma and weight gain^(115,133)^. In some of the included studies in the current review, there was mention of unhealthy weight control methods such as the using slimming tablets and smoking, and obesity was associated with negative attributes and stigmatisation. These findings imply an urgent need for interventions that increase understanding of healthy weight limits, provide opportunities for weight screening in communities and support healthy weight control. However, there is a need to be aware that increased knowledge of excess weight may increase stigma, necessitating mitigation efforts as stigma may result in further weight gain^(115)^.

Previous reviews have suggested that high body weight is preferred in SSA, especially among women^(11,134)^. However, these reviews did not explore the estimated ideal body weight. The current review found that the ideal body weight for most people was between the midpoint of normal weight and upper limit of overweight. The widespread view of overweight as the ideal body weight in SSA may be due to a need to strike a balance between not being thin or too fat, as being thin may be associated with poor health^(135)^ or poverty^(37,91)^, while being too fat may be perceived negatively^(37,39,79)^. Traditionally, the need to counter the negative perceptions associated with lean bodies may have resulted to obesity being positively perceived. However, the current review demonstrated that positive perceptions of obesity are being challenged: with lean bodies being viewed as attractive^(38,39,41)^ and not associated with poor health^(136)^ or poverty^(38,92)^, and an increased understanding of the health implications of obesity^(38,40–42,80,136)^. Nevertheless, the fact that overweight is seen as an ideal body weight still presents increased risk, as type 2 diabetes develops at low body weights in SSA than high-income settings^(137)^. Additionally, central obesity is a better predictor of diabetes than general obesity in SSA^(138–146)^. This suggests that interventions focusing on the reduction of central obesity would be more effective in reducing diabetes risk than those focusing on general weight loss. The success of such interventions could be supported by women’s preferences for *‘maintaining a flat stomach’*, as reported in Ghana^(79)^; however, they might be undermined by men’s preferences for an *‘administrative belly’, ‘executive belly’ or ‘commanding belly’*, as reported in Cameroon^(40,114)^. Therefore, there is an urgent need to educate and persuade people, especially men, about the risks associated with high central obesity, and support them to lose weight.

Similar to weight, most people knew that unhealthy diets (especially high sugar intake) were a diabetes risk factor. Nevertheless, there was limited understanding of the role of excess energy rather than simply sugar intake, as reported in Cameroon^(40)^, in the development of diabetes and of the protective effect of fruit and vegetables, as reported in Senegal^(93)^. Furthermore, knowledge of high sugar intake as a diabetes risk factor did not translate to better dietary practices. For instance, in studies that explored dietary practices (mainly from South Africa), sugar intake was high. Taken together these findings imply a need for improved knowledge of the role of excess energy rather than just sugar intake in diabetes risk and of fruit and vegetable intake in diabetes prevention. Nevertheless, knowledge about diabetes risk may not necessarily result in better dietary practices, and thus it is essential to understand the barriers to healthy eating. In this review, the most commonly reported barriers were as follows: the limited availability and affordability of healthy foods, the availability and accessibility of Western diets; and unhealthy traditional dietary practices. Therefore, efforts to promote healthy eating need to complement increasing knowledge at the individual level with interventions on other areas of influence such as increasing access of healthy foods within the physical environment, changing social norms and modelling healthy eating within the social environment, and implementing policies that ensure food security and the affordability (e.g. subsidised) of healthy foods and restrict access to unhealthy foods at the macro-level^(9,147,148)^.

Many people reported engaging in regular physical activity. Evidence from surveys in twenty-two countries suggests that the majority of adults (>75 %) in SSA meet the WHO physical activity recommendations, mainly through travel- and work-related physical activity^(149)^. However, there is evidence that physical activity levels are declining with increasing economic development and urbanisation: for example, the physical activity levels have reduced over time among school-aged children in SSA, especially those in urban settings^(7)^. Consequently, there is a need to explore barriers to engaging in physical activity (especially leisure-time physical activity) to inform diabetes prevention initiatives. In the current review, two of the main reported barriers were the limited availability and affordability of physical activity facilities and perceived time constraints. This is consistent with reviews in high-income settings which found that lack of facilities among African American women^(150)^ and time constraints among inactive Australia adults^(151)^ are as important barriers to physical activity. This finding suggests that interventions are needed to increase the availability and affordability of physical activity facilities, such as the establishment of community gymnasiums. Apart from facilities, educating people about simple types of leisure physical activity, such as walking, may also help people to increase and/or maintain their physical activity levels. Furthermore, to overcome time constraints, people can be encouraged to fit physical activity into their daily routines, for example, through active travel and/or home exercising^(148)^.

Strengths of our review included using seven databases to identify and synthesise extensive evidence from both quantitative and qualitative studies on knowledge, perceptions and practices related to diabetes risk in SSA over 20 years allowed for a comprehensive account of current evidence. Nevertheless, only nineteen countries were represented in included studies, and most studies were from South Africa, which limits the generalisability of our findings to the SSA region. Furthermore, this review focused on weight, diet, and physical activity and did not include other known diabetes risk factors such as stress and substance use, which emerged as areas of concern in a recent citizen science study conducted in four SSA countries^(152)^. A potential limitation was the exclusion of articles that were not written in English: although this may have led to missing some articles from Francophone SSA, it is important to note that fourteen articles from six Francophone countries (Cameroon, Côte d’Ivoire, Rwanda, Madagascar, Seychelles and Senegal) were included in the review.

In conclusion, most people in SSA appear to be broadly aware of the three main risk factors associated with type 2 diabetes (excess weight, unhealthy diet and physical inactivity). However, lack of specific knowledge of healthy weight limits and importance of eating fruit and vegetables may contribute to people with excess weight underestimating their weight, not engaging in weight control and not eating enough fruit and vegetables. Important perceived barriers to lifestyle modification include social (e.g. societal influences promoting weight gain) and environmental (unavailability and/or unaffordability of healthy foods and physical activity facilities) barriers. Our findings highlight the need for multicomponent diabetes prevention interventions that increase detailed knowledge about diabetes risk (e.g. healthy weight limits and what constitutes a healthy diet) at an individual level and create social (e.g. societal perceptions that promote healthy living) and physical (e.g. increased availability and affordability of healthy foods and physical activity facilities, and restricting access to unhealthy foods) environments to support healthy lifestyles. Finally, there is a need for more research on experiences of diabetes risk to be undertaken outside of South Africa.

## Supporting information

Manyara et al. supplementary material 1 Manyara et al. supplementary material

Manyara et al. supplementary material 2 Manyara et al. supplementary material

Manyara et al. supplementary material 3 Manyara et al. supplementary material

## Supplementary material

For supplementary material accompanying this paper visit https://doi.org/10.1017/S1368980024000752
