# Supplementary material for: Knowledge, perceptions and practices towards diabetes risk in sub-Saharan Africa: a mixed-methods scoping review
Source: Public Health Nutr. 2024 Mar 27;27(1):e104. doi: 10.1017/S1368980024000752 (PMC11010065; doi:10.1017/S1368980024000752)
Supplement: Manyara et al. supplementary material 2 — Manyara et al. supplementary material [file S1368980024000752sup002.docx]

# PubMed

| Search | Query |
| --- | --- |
| #27 | Search ((#11) AND #24) AND #26 Filters: Publication date from 2000/01/01 to 2019/12/31; Humans Sort by: [pubsolr12]  **Search updated in March 2021 and October 2023** |
| #26 | Search "Africa"[Mesh] OR "Africa South of the Sahara"[Mesh]OR ( "Congo"[Mesh] OR "Tanzania"[Mesh] OR "Guinea-Bissau"[Mesh] OR "Eritrea"[Mesh] OR "Comoros"[Mesh] OR "Equatorial Guinea"[Mesh] OR "Zimbabwe"[Mesh] OR "Zambia"[Mesh] OR "Uganda"[Mesh] OR "Togo"[Mesh] OR "Swaziland"[Mesh] OR "Sudan"[Mesh] OR "South Africa"[Mesh] OR "Somalia"[Mesh] OR "Sierra Leone"[Mesh] OR "Senegal"[Mesh] OR "Rwanda"[Mesh] OR "Nigeria"[Mesh] OR "Niger"[Mesh] OR "Namibia"[Mesh] OR "Mozambique"[Mesh] OR "Mauritania"[Mesh] OR "Mali"[Mesh] OR "Malawi"[Mesh] OR "Madagascar"[Mesh] OR "Liberia"[Mesh] OR "Lesotho"[Mesh] OR "Kenya"[Mesh] OR "Cote d'Ivoire"[Mesh] OR "Ghana"[Mesh] OR "Gambia"[Mesh] OR "Gabon"[Mesh] OR "Ethiopia"[Mesh] OR "Chad"[Mesh] OR "Central African Republic"[Mesh] OR "Cameroon"[Mesh] OR "Burundi"[Mesh] OR "Burkina Faso"[Mesh] OR "Botswana"[Mesh] OR "Benin"[Mesh] OR "Angola"[Mesh] OR "Sao Tome and Principe"[Mesh] OR "Democratic Republic of the Congo"[Mesh] Filters: Publication date from 2000/01/01 to 2019/12/31; Humans Sort by: [pubsolr12] |
| #24 | Search (((((((("Diabetes Mellitus"[Mesh]) OR ( "Body Weight"[Mesh] OR "Ideal Body Weight"[Mesh] OR "Weight Loss"[Mesh] OR "Weight Gain"[Mesh] )) OR "Body Size"[Majr]) OR "Overweight"[Mesh]) OR "Obesity"[Mesh]) OR ( "Diet"[Mesh] OR "Diet, Western"[Mesh] OR "Diet, Mediterranean"[Mesh] OR "Healthy Diet"[Mesh] )) OR ( "Food"[Mesh] OR "Food Quality"[Mesh] )) OR "Exercise"[Mesh]) OR "Sedentary Behavior"[Mesh] Filters: Publication date from 2000/01/01 to 2019/12/31; Humans Sort by: [pubsolr12] |
| #11 | Search (((((("Knowledge"[Mesh] OR "Health Knowledge, Attitudes, Practice"[Mesh]) OR "Attitude to Health"[Mesh]) OR "Awareness"[Mesh]) OR "Perception"[Mesh]) OR "Culture"[Mesh]) OR "Comprehension"[Mesh]) OR "Health Behavior"[Mesh] Filters: Publication date from 2000/01/01 to 2019/12/31; Humans Sort by: [pubsolr12] |

# MEDLINE Ovid


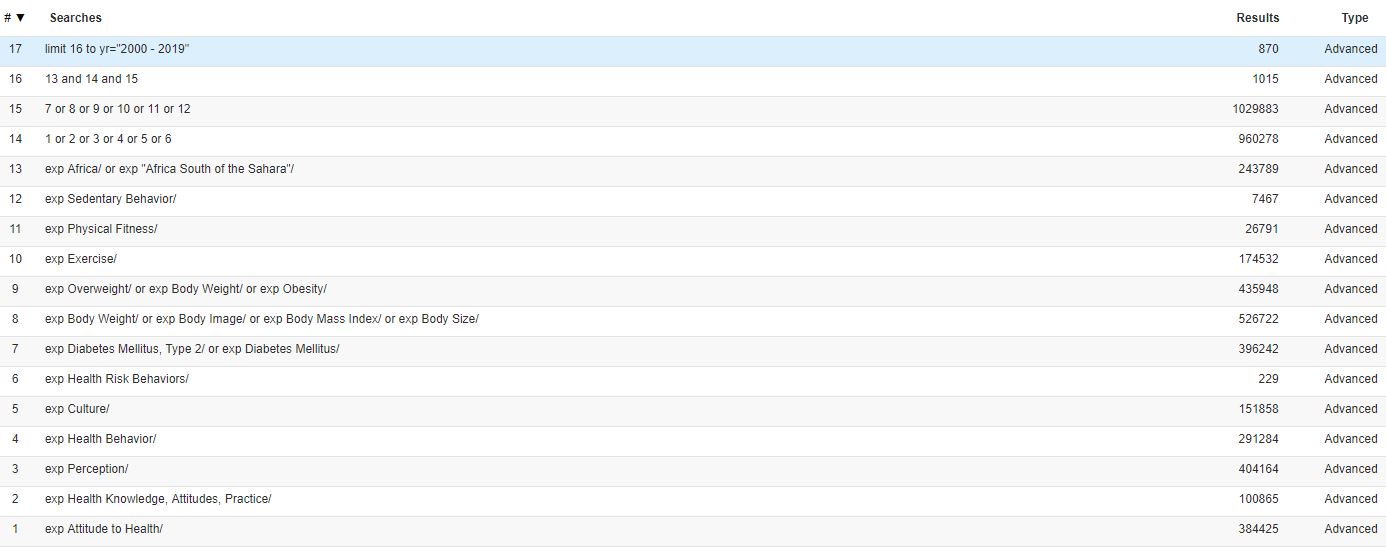


# Web of Science

**Search updated in March 2021 and October 2023**


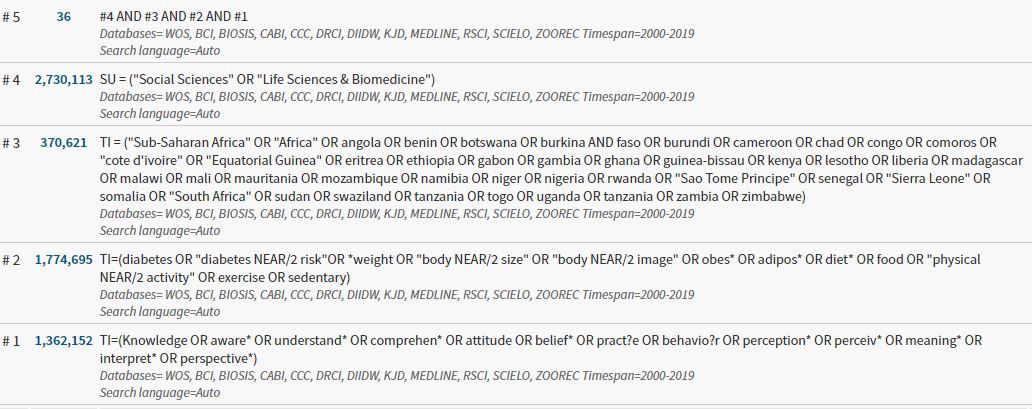


# Scopus

( ( TITLE-ABS-KEY ( knowledge OR aware* OR understand* OR comprehen* OR attitude OR belief* OR practi?e OR behavio?r OR perception* OR perceiv* OR meaning* OR intrepret* OR perspective* ) AND PUBYEAR > 2000 ) AND ( TITLE-ABS-KEY ( diabetes OR "diabetes risk" *weight OR "body W/2 size" OR "body W/2 image" obes* OR adipos* OR diet OR food OR "physical activity" OR exercise OR sedentary ) AND PUBYEAR > 2000 ) ) AND ( TITLE-ABS-KEY ( africa OR "Sub-Saharan Africa" OR angola OR benin OR botswana OR "burkina faso" OR burundi OR cameroon OR chad OR congo OR comoros OR "cote d'ivoire" OR "Equatorial Guinea" OR eritrea OR ethiopia OR gabon OR gambia OR ghana OR guinea-bissau OR kenya OR lesotho OR liberia OR madagascar OR malawi OR mali OR mauritania OR mozambique OR namibia OR niger OR nigeria OR rwanda OR "Sao Tome Principe" OR senegal OR "Sierra Leone" OR somalia OR "South Africa" OR sudan OR swaziland OR tanzania OR togo OR uganda OR tanzania OR zambia OR zimbabwe ) AND PUBYEAR > 2000 ) AND ( LIMIT-TO ( SUBJAREA , "MEDI" ) OR LIMIT-TO ( SUBJAREA , "SOCI" ) OR LIMIT-TO ( SUBJAREA , "NURS" ) OR EXCLUDE ( SUBJAREA , "ARTS" ) OR EXCLUDE ( SUBJAREA , "PSYC" ) OR EXCLUDE ( SUBJAREA , "BIOC" ) OR EXCLUDE ( SUBJAREA , "NURS" ) OR EXCLUDE ( SUBJAREA , "NEUR" ) OR EXCLUDE ( SUBJAREA , "ENVI" ) OR EXCLUDE ( SUBJAREA , "BUSI" ) ) AND ( LIMIT-TO ( DOCTYPE , "ar" ) ) AND ( LIMIT-TO ( LANGUAGE , "English" ) )

# CINAHL EBSCOhost


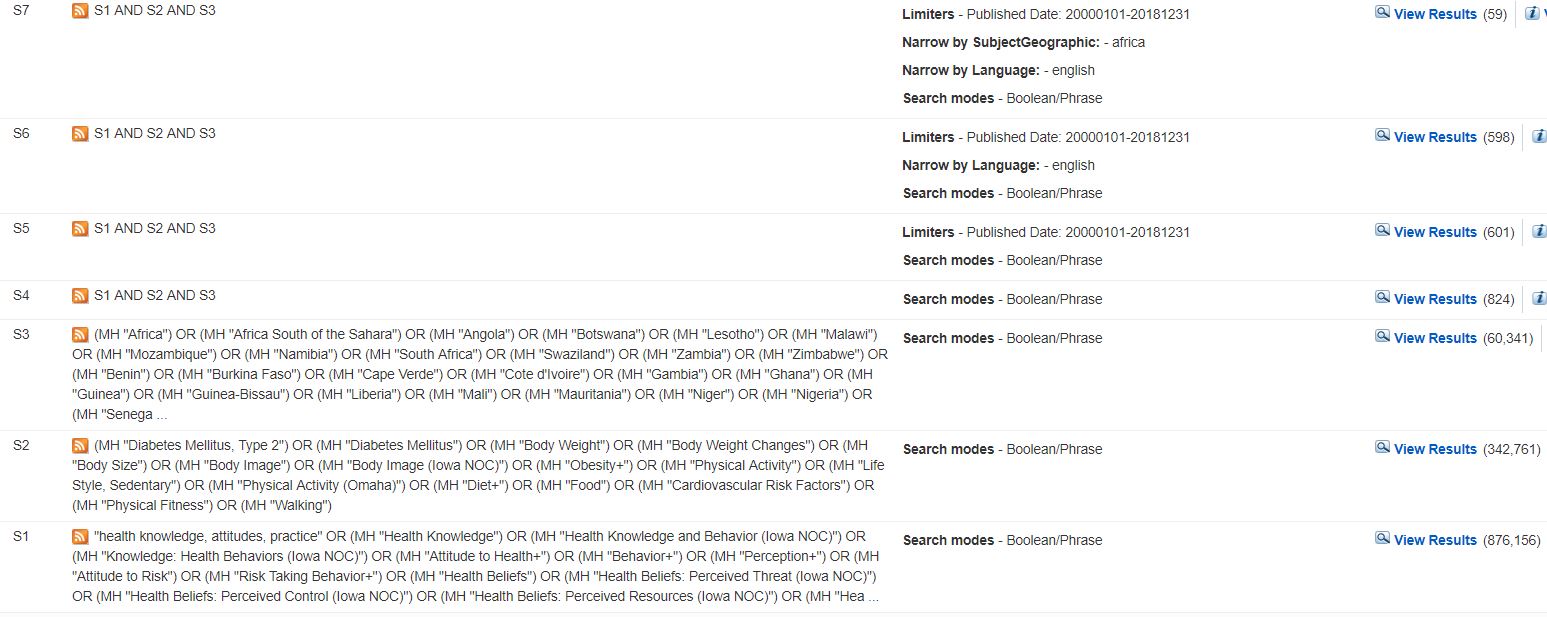


# PsychINFO EBSCOhost


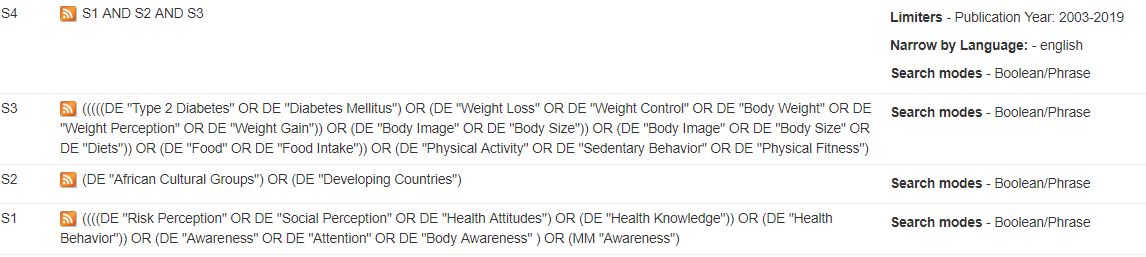


# Africa Journals Online

| **Advanced Search of keywords** | **Comment** |
| --- | --- |
| knowledge OR attitude OR practice OR perception AND "diabetes risk"  knowledge OR attitude OR practice OR perception AND diet  knowledge OR attitude OR practice OR perception AND weight  knowledge OR attitude OR practice OR perception AND "physical activity" | Given the advanced search resulted to only two articles a simple search on the title was done |
| **Simple search on title** |  |
| “diabetes risk” | Due lack of a way to export search results to Endnote, titles were screened on the database and only relevant titles were searched on Google for export to Endnote. 22 article titles were exported |
| Physical activity |  |
| Body weight |  |
| diet |  |
