# Supplementary material for: Knowledge, perceptions and practices towards diabetes risk in sub-Saharan Africa: a mixed-methods scoping review
Source: Public Health Nutr. 2024 Mar 27;27(1):e104. doi: 10.1017/S1368980024000752 (PMC11010065; doi:10.1017/S1368980024000752)
Supplement: Manyara et al. supplementary material 3 — Manyara et al. supplementary material [file S1368980024000752sup003.docx]

# Table 1: List of included articles

| Author and year | Country | Study design | Knowledge of diabetes risk factors | Weight perceptions/practices | Diet perceptions/practices | Physical activity perceptions/practices |
| --- | --- | --- | --- | --- | --- | --- |
| 1. Adedokun et al 2019^(1)^ | South Africa | Cross-sectional study |  |  | √ | √ |
| 1. Agbana et al 2020^(2)^ | Nigeria | Cross-sectional study | √ |  | √ | √ |
| 1. Akinpelu et al 2015^(3)^ | Nigeria | Cross-sectional study |  | √ |  |  |
| 1. Alemayehu et al 2020^(4)^ | Ethiopia | Cross-sectional study | √ |  |  |  |
| 1. Alemayehu and Sisay 2021^(5)^ | Ethiopia | Cross-sectional study | √ |  |  |  |
| 1. Alwan et al 2010^(6)^ | Seychelles | Cross-sectional study |  | √ |  |  |
| 1. Appiah et al 2016^(7)^ | Ghana | Cross-sectional study |  | √ |  |  |
| 1. Aryeety 2016^(8)^ | Ghana | Qualitative |  | √ |  |  |
| 1. Asante et al 2023^(9)^ | Ghana | Cross-sectional study | √ |  |  | √ |
| 1. Asmamaw et al 2015^(10)^ | Ethiopia | Cross-sectional study | √ |  |  |  |
| 1. Awosan et al 2017^(11)^ | Nigeria | Cross-sectional study |  | √ |  |  |
| 1. Balis et al. 2019^(12)^ | Ghana | Mixed methods |  |  |  | √ |
| 1. Balla et al 2013^(13)^ | Sudan | Cross-sectional study | √ |  |  |  |
| 1. Benkeser et al 2012^(14)^ | Ghana | Cross-sectional study |  | √ |  |  |
| 1. Bosire et al 2020^(15)^ | South Africa | Qualitative |  |  | √ |  |
| 1. Bosire et al 2020^(16)^ | South Africa | Qualitative |  | √ |  |  |
| 1. Chigbu et al 2021^(17)^ | Nigeria | Cross-sectional study |  | √ |  |  |
| 1. Cohen et al 2013^(18)^ | Cameroon | Mixed methods |  | √ |  |  |
| 1. Cohen et al 2017^(19)^ | Cameroon | Mixed Methods |  | √ | √ | √ |
| 1. Cohen et al 2019^(20)^ | South Africa | Cross-sectional study |  | √ |  |  |
| 1. Cohen et al 2019^(21)^ | Senegal | Mixed methods |  | √ |  |  |
| 1. Dada et al 2021^(22)^ | Nigeria | Cross-sectional study |  | √ | √ |  |
| 1. Dolman et al 2007^(23)^ | South Africa | Cross-sectional study |  |  | √ |  |
| 1. Draper et al 2016^(24)^ | South Africa | Qualitative |  | √ |  |  |
| 1. Duda et al 2006^(25)^ | Ghana | Cross-sectional study |  | √ |  |  |
| 1. Duda et al 2007^(26)^ | Ghana | Cross-sectional study |  | √ |  |  |
| 1. Ejike 2015^(27)^ | Nigeria | Cross-sectional study |  | √ |  |  |
| 1. Ettarh et al 2013^(28)^ | Kenya | Cross-sectional study |  | √ |  |  |
| 1. Faber et al 2005^(29)^ | South Africa | Cross-sectional study |  | √ |  |  |
| 1. Fezeu et al 2010^(30)^ | Cameroon | Cross-sectional study | √ |  |  |  |
| 1. Gatoa et al 2017^(31)^ | Ghana | Cross-sectional study |  |  | √ |  |
| 1. Gebremariam et al 2018^(32)^ | Ethiopia | Qualitative |  |  | √ |  |
| 1. Govender 2018^(33)^ | South Africa | Cross-sectional study |  | √ |  |  |
| 1. Hill et al 2020^(34)^ | South Africa | Mixed methods |  |  |  | √ |
| 1. Holdsworth et al 2004^(35)^ | Senegal | Cross-sectional study |  | √ |  |  |
| 1. Holdsworth et al 2006^(36)^ | Senegal | Cross-sectional study |  |  | √ |  |
| 1. Hughes et al 2006^(37)^ | South Africa | Mixed Methods | √ |  |  |  |
| 1. Hunter-Adams 2019^(38)^ | South Africa | Qualitative |  | √ |  |  |
| 1. Iliyasu et al 2013^(39)^ | Nigeria | Cross-sectional study |  | √ |  |  |
| 1. Jumah and Duda 2007^(40)^ | Ghana | Cross-sectional study |  | √ |  |  |
| 1. Kanozire and Pretorius 2023^(41)^ | South Africa | Cross-sectional study |  | √ |  |  |
| 1. Kassahun and Mekonen 2017^(42)^ | Ethiopia | Cross-sectional study | √ |  |  |  |
| 1. Kiawi et al 2006^(43)^ | Cameroon | Qualitative | √ |  |  |  |
| 1. Kiguli et al 2019^(44)^ | Uganda | Qualitative |  |  | √ |  |
| 1. Macia et al 2017^(45)^ | Senegal | Cross-sectional study |  | √ |  |  |
| 1. Maina et al 2010^(46)^ | Kenya | Cross-sectional study | √ |  |  |  |
| 1. Malete et al 2022^(47)^ | Botswana | Cross-sectional study |  |  |  | √ |
| 1. Manafe et al 2022^(48)^ | South Africa | Qualitative |  | √ |  |  |
| 1. Maruf et al 2012^(49)^ | Nigeria | Cross-sectional study |  | √ |  |  |
| 1. Mayega et al 2014^(50)^ | Uganda | Qualitative | √ |  |  |  |
| 1. Mchiza et al 2015^(51)^ | South Africa | Cross-sectional study |  | √ |  |  |
| 1. Mogre et al 2013^(52)^ | Ghana | Cross-sectional study |  | √ |  |  |
| 1. Mogre et al 2015^(53)^ | Ghana | Cross-sectional study |  | √ |  |  |
| 1. Muhihi 2012^(54)^ | Tanzania | Cross-sectional study |  | √ |  |  |
| 1. Mukeshimana and Nkosi 2013^(55)^ | Rwanda | Cross-sectional study | √ |  |  |  |
| 1. Muzindutsi et al 2014^(56)^ | South Africa | Cross-sectional study |  |  |  | √ |
| 1. Nambaka et al 2011^(57)^ | Kenya | Cross-sectional study |  |  |  | √ |
| 1. Nannseu et al 2019^(58)^ | Cameroon | Cross-sectional study | √ |  |  |  |
| 1. Ndambo et al 2022^(59)^ | Malawi | Qualitative |  | √ | √ |  |
| 1. Ndejjo et al 2022^(60)^ | Uganda | Qualitative | √ |  | √ | √ |
| 1. Nizeyimana and Phillips 2006^(61)^ | Uganda | Cross-sectional study |  |  |  | √ |
| 1. Nolan and Surujlal 2011^(62)^ | South Africa | Cross-sectional study |  |  |  | √ |
| 1. Nolan et al 2011^(63)^ | South Africa | Cross-sectional study |  |  |  | √ |
| 1. Odukoya et al 2022^(64)^ | Nigeria | Qualitative |  |  | √ |  |
| 1. Ojofeitimi et al 2007^(65)^ | Nigeria | Cross-sectional study |  | √ |  |  |
| 1. Okeke et al 2006^(66)^ | Nigeria | Cross-sectional study |  | √ |  |  |
| 1. Okop et al 2016^(67)^ | South Africa | Qualitative |  | √ |  |  |
| 1. Okop et al 2019^(68)^ | South Africa | Cross-sectional study |  | √ |  |  |
| 1. Okoro et al 2014^(69)^ | Nigeria | Cross-sectional study |  | √ |  |  |
| 1. Olaoye and Oyetunde 2012^(70)^ | Nigeria | Cross-sectional study |  | √ |  |  |
| 1. Osiberu 2021^(71)^ | Nigeria | Cross-sectional study | √ |  |  |  |
| 1. Oyeyemi et al 2011^(72)^ | Nigeria | Cross-sectional study |  |  |  | √ |
| 1. Peltzer 2001^(73)^ | South Africa | Cross-sectional study |  |  | √ |  |
| 1. Peltzer 2002^(74)^ | South Africa | Cross-sectional study |  |  | √ |  |
| 1. Peltzer 2002^(75)^ | South Africa | Cross-sectional study |  |  | √ |  |
| 1. Peltzer 2003^(76)^ | South Africa | Cross-sectional study |  |  |  | √ |
| 1. Peltzer 2004^(77)^ | South Africa | Cross-sectional study |  |  | √ |  |
| 1. Peltzer and Pengpid 2006^(78)^ | South Africa | Cross-sectional study |  |  |  | √ |
| 1. Peltzer and Pengpid 2012^(79)^ | South Africa | Cross-sectional study |  | √ |  |  |
| 1. Peltzer and Pengpid 2015^(80)^ | Multinational - Ivory Coast, Madagascar, Mauritius, Namibia, Nigeria, South Africa | Cross-sectional study |  | √ |  |  |
| 1. Peltzer and Promtussananon 2004^(81)^ | South Africa | Cross-sectional study |  |  | √ |  |
| 1. Phetla et al 2017^(82)^ | South Africa | Cross-sectional study |  | √ |  |  |
| 1. Phillips et al 2016^(83)^ | South Africa | Qualitative |  | √ | √ | √ |
| 1. Prioreschi et al 2017^(84)^ | South Africa | Cross-sectional study |  | √ |  |  |
| 1. Prioreschi et al 2021^(85)^ | South Africa | Cross-sectional study |  |  | √ | √ |
| 1. Puoane et al 2005^(86)^ | South Africa | Mixed Methods |  | √ |  |  |
| 1. Puoane et al 2006^(87)^ | South Africa | Qualitative |  | √ | √ |  |
| 1. Ramautar et al 2021^(88)^ | South Africa | Cros-sectional study |  |  |  | √ |
| 1. Rutebemberwa et al 2013^(89)^ | Uganda | Qualitative | √ |  |  |  |
| 1. Shiferaw et al 2020^(90)^ | Ethiopia | Cross-sectional study | √ |  |  |  |
| 1. Siervo et al 2006^(91)^ | Gambia | Cross-sectional study |  | √ |  |  |
| 1. Simfukwe et al 2017^(92)^ | South Africa | Qualitative |  | √ |  |  |
| 1. Skaal and Pengpid 2011^(93)^ | South Africa | Cross-sectional study |  | √ |  |  |
| 1. Tuakli-Wosornu et al 2014^(94)^ | Ghana | Mixed Methods |  |  |  | √ |
| 1. Tumusiime and Frantz 2006^(95)^ | Rwanda | Cross-sectional study |  |  |  | √ |
| 1. Tuoyire et al 2018^(96)^ | Ghana | Qualitative |  | √ |  |  |
| 1. van den Berg et al 2012^(97)^ | South Africa | Cross-sectional study |  |  | √ |  |
| 1. Venter et al 2009^(98)^ | South Africa | Cross-sectional study |  | √ |  |  |
| 1. Walter et al 2011^(99)^ | South Africa | Qualitative |  |  |  | √ |
| 1. Yepes et al 2016^(100)^ | Seychelles | Cross-sectional study |  | √ |  |  |
| 1. Yiga et al 2021^(101)^ | Uganda | Qualitative |  |  | √ | √ |

1. Adedokun AO, Ter Goon D, Owolabi EO *et al.* (2019) Prevalence, awareness, and determinants of type 2 diabetes mellitus among commercial taxi drivers in buffalo city metropolitan municipality South Africa: A cross-sectional survey. *Medicine* 98, e14652.

2. Agbana RD, Adegbilero-Iwari OE, Amu EO Ijabadeniyi OA (2020) Awareness and risk burden of diabetes mellitus in a rural community of Ekiti State, South-Western Nigeria. *J Prev Med Hyg* 61, E593-e600.

3. Akinpelu AO, OO: Adekanla, BA (2015) Body size perceptions and weight status of adults in a Nigerian rural community. *Annals of medical and health sciences research* 5, 358-364.

4. Alemayehu AM, Dagne H Dagnew B (2020) Knowledge and associated factors towards diabetes mellitus among adult non-diabetic community members of Gondar city, Ethiopia 2019. *PloS one* 15, e0230880.

5. Alemayehu AM & Sisay MM (2021) Attitude towards diabetes mellitus among adult communities in Gondar city, Ethiopia. *PLoS One* 16, e0251777.

6. Alwan H, Viswanathan B, Williams J *et al.* (2010) Association between weight perception and socioeconomic status among adults in the Seychelles. *BMC public health* 10, 467.

7. Collins AA, Gloria EO Matilda S-A (2016) Preferred body size in urban Ghanaian women: implication on the overweight/obesity problem. *PAMJ* 23.

8. Aryeetey RN (2016) Perceptions and Experiences of Overweight among Women in the Ga East District, Ghana. *Front Nutr* 3, 13.

9. Asante DO, Dai A, Walker AN *et al.* (2023) Assessing hypertension and diabetes knowledge, attitudes and practices among residents in Akatsi South District, Ghana using the KAP questionnaire. *Front Public Health* 11, 1056999.

10. Asmamaw A, Asres G, Negese D *et al.* (2015) Knowledge and attitude about diabetes mellitus and its associated factors among people in Debre Tabor town, Northwest Ethiopia: cross sectional study. *Science* 3, 199-209.

11. Awosan KJA, S. A.: Bello, H.: Bello-Ibrahim, Z. (2017) Nutritional status, weight perception and weight control practices among office employees in Sokoto, Nigeria. *The Pan African medical journal* 27, 279.

12. Balis LE, Sowatey G, Ansong-Gyimah K *et al.* (2019) Older Ghanaian adults' perceptions of physical activity: an exploratory, mixed methods study. *BMC geriatrics* 19, 85.

13. Balla SA, Ahmed HA Awadelkareem MA (2014) Prevalence of diabetes, knowledge, and attitude of rural, population towards diabetes and hypoglycaemic event, Sudan 2013. *Am J Health Res* 2, 356-360.

14. Benkeser RMB, R.: Hill, A. G. (2012) Prevalence of overweight and obesity and perception of healthy and desirable body size in urban, Ghanaian women. *Ghana Medical Journal* 46, 66-75.

15. Bosire EN, Cohen E, Erzse A *et al.* (2020) 'I'd say I'm fat, I'm not obese': obesity normalisation in urban-poor South Africa. *Public health nutrition* 23, 1515-1526.

16. Bosire EN, Stacey N, Mukoma G *et al.* (2020) Attitudes and perceptions among urban South Africans towards sugar-sweetened beverages and taxation. *Public health nutrition* 23, 374-383.

17. Chigbu CO, Aniebue UU, Berger U Parhofer KG (2021) Impact of perceptions of body size on obesity and weight management behaviour: a large representative population study in an African setting. *Journal of Public Health* 43, e54-e61.

18. Cohen EB, G.: Palstra, F. P.: Pasquet, P. (2013) Social valorisation of stoutness as a determinant of obesity in the context of nutritional transition in Cameroon: the Bamileke case. *Social Science & Medicine* 96, 24-32.

19. Cohen EA, N.: Ponty, A.: Loinger-Beck, J.: Nkuintchua, T.: Monteillet, N.: Bernard, J. Y.: Said-Mohamed, R.: Holdsworth, M.: Pasquet, P. (2017) Nutrition Transition and Biocultural Determinants of Obesity among Cameroonian Migrants in Urban Cameroon and France. *International Journal of Environmental Research & Public Health [Electronic Resource]* 14, 29.

20. Cohen E, Gradidge PJ, Micklesfield LK Norris SA (2019) Relationship Between Body Mass Index and Body Image Disturbances Among South African Mothers and Their Daughters Living in Soweto, Johannesburg. *Family & community health* 42, 140-149.

21. Cohen E, Gradidge PJ, Ndao A *et al.* (2019) Biocultural determinants of overweight and obesity in the context of nutrition transition in Senegal: a holistic anthropological approach. *Journal of biosocial science* 51, 469-490.

22. Dada SO, Oyewole OE Desmennu AT (2021) Knowledge as Determinant of Healthy-Eating Among Male Postgraduate Public Health Students in a Nigerian Tertiary Institution. *International Quarterly of Community Health Education* 42, 103-114.

23. Dolman RCS, W.: van't Riet, H.: Badham, J.: Jerling, J. C. (2008) Beliefs of South Africans regarding food and cardiovascular health. *Public Health Nutrition* 11, 946-954.

24. Draper CED, K. J.: Goedecke, J. H. (2016) Perceptions relating to body size, weight loss and weight-loss interventions in black South African women: a qualitative study. *Public Health Nutrition* 19, 548-556.

25. Duda RBJ, N. A.: Hill, A. G.: Seffah, J.: Biritwum, R. (2006) Interest in healthy living outweighs presumed cultural norms for obesity for Ghanaian women. *Health and Quality of Life Outcomes* 4.

26. Duda RBJ, N. A.: Hill, A. G.: Seffah, J.: Biritwum, R. (2007) Assessment of the ideal body image of women in Accra, Ghana. *Tropical Doctor* 37, 241-244.

27. Ejike CE (2015) Body shape dissatisfaction is a 'normative discontent' in a young-adult Nigerian population: A study of prevalence and effects on health-related quality of life. *Journal of Epidemiology and Global Health* 5, S19-26.

28. Ettarh RVdV, S.: Oti, S.: Kyobutungi, C. (2013) Overweight, obesity, and perception of body image among slum residents in Nairobi, Kenya, 2008-2009. *Preventing Chronic Disease* 10, E212.

29. Faber MK, H. S. (2005) Dietary intake, perceptions regarding body weight, and attitudes toward weight control of normal weight, overweight, and obese Black females in a rural village in South Africa. *Ethn Dis* 15, 238-245.

30. Fezeu LF, E.: Ngufor, G.: Mbeh, G.: Mbanya, J. C. (2010) Diabetes awareness in general population in Cameroon. *Diabetes Research & Clinical Practice* 90, 312-318.

31. Gatoa WEA, S.: Apentengc, B. A.: Opokuc, S. T.: Boakyed, B. K. (2017) Diabetes in the Cape Coast metropolis of Ghana: An assessment of risk factors, nutritional practices and lifestyle changes. *International Health* 9, 310-316.

32. Gebremariam LWA, A.: Kahsay, A. B.: Hirakawa, Y.: Chiang, C.: Yatsuya, H.: Matsuyama, A. (2018) Perception and practice of 'healthy' diet in relation to noncommunicable diseases among the urban and rural people in northern Ethiopia: a community-based qualitative study. *Nagoya J Med Sci* 80, 451-464.

33. Govender RD, Al-Shamsi S Regmi D (2019) Weight bias and eating behaviours of persons with overweight and obesity attending a general medical practice in Durban, South Africa. *South African Family Practice* 61, 85-90.

34. Hill J, Lavigne Delville C, Auorousseau AM *et al.* (2020) Development of a Tool to Increase Physical Activity among People at Risk for Diabetes in Low-Resourced Communities in Cape Town. *International Journal of Environmental Research & Public Health [Electronic Resource]* 17, 30.

35. Holdsworth MG, A.: Landais, E.: Maire, B.: Delpeuch, F. (2004) Perceptions of healthy and desirable body size in urban Senegalese women. *International Journal of Obesity & Related Metabolic Disorders: Journal of the International Association for the Study of Obesity* 28, 1561-1568.

36. Holdsworth MD, F.: Landais, E.: Gartner, A.: Eymard-Duvernay, S.: Maire, B. (2006) Knowledge of dietary and behaviour-related determinants of non-communicable disease in urban Senegalese women. *Public Health Nutrition* 9, 975-981.

37. Hughes GD, Puoane T Bradley H (2006) Ability to manage diabetes—community health workers' knowledge, attitudes and beliefs. *Journal of Endocrinology, Metabolism and Diabetes of South Africa* 11, 10-14.

38. Hunter-Adams J (2019) Perceptions of weight in relation to health, hunger, and belonging among women in periurban South Africa. *Health care for women international* 40, 347-364.

39. Iliyasu ZA, I. S.: Abubakar, S.: Lawan, U. M.: Gajida, A. U.: Jibo, A. M. (2013) A survey of weight perception and social desirability of obesity among adults in Kano Metropolis, Northern Nigeria. *Niger J Med* 22, 101-108.

40. Jumah NA & Duda RB (2007) Comparison of the perception of ideal body images of Ghanaian men and women. *African Journal of Health Sciences* 14, 54-60.

41. Kanozire B & Pretorius D (2023) Obese patients' dissatisfaction with weight, body image and clinicians' interaction at a district hospital; Gauteng. *Afr* 15, e1-e9.

42. Kassahun CWM, A. G. (2017) Knowledge, attitude, practices and their associated factors towards diabetes mellitus among non diabetes community members of Bale Zone administrative towns, South East Ethiopia. A cross-sectional study. *PLoS One* 12, e0170040.

43. Kiawi EE, R.: Shu, J.: Unwin, N.: Kamadjeu, R.: Mbanya, J. C. (2006) Knowledge, attitudes, and behavior relating to diabetes and its main risk factors among urban residents in Cameroon: a qualitative survey. *Ethn Dis* 16, 503-509.

44. Kiguli J, Alvesson HM, Mayega RW *et al.* (2019) Dietary patterns and practices in rural eastern Uganda: Implications for prevention and management of type 2 diabetes. *Appetite* 143, 104409.

45. Macia EC, E.: Gueye, L.: Boetsch, G.: Duboz, P. (2017) Prevalence of obesity and body size perceptions in urban and rural Senegal: new insight on the epidemiological transition in West Africa. *Cardiovasc J Afr* 28, 324-330.

46. Kiberenge MWN, Z. M.: Njenga, E. W.: Muchemi, E. W. (2010) Knowledge, attitude and practices related to diabetes among community members in four provinces in Kenya: a cross-sectional study. *Pan Afr Med J* 7, 2.

47. Malete L, Ricketts C, Chen S Jackson J (2022) Correlates of Physical Activity Among Adults in Botswana: Sociodemographic Factors, Health Status, and Body Image. *J Phys Act Health* 19, 599-606.

48. Manafe M, Chelule PK Madiba S (2022) The Perception of Overweight and Obesity among South African Adults: Implications for Intervention Strategies. *International Journal of Environmental Research & Public Health [Electronic Resource]* 19, 28.

49. Maruf FA, Akinpelu AO Nwankwo MJ (2012) Perceived body image and weight: discrepancies and gender differences among University undergraduates. *African health sciences* 12, 464-472.

50. Mayega RWE, S.: Rutebemberwa, E.: Tomson, G.: Kiguli, J. (2014) 'Change means sacrificing a good life': perceptions about severity of type 2 diabetes and preventive lifestyles among people afflicted or at high risk of type 2 diabetes in Iganga Uganda. *BMC Public Health* 14, 864.

51. McHiza ZJP, W. A.: Makoae, M.: Sewpaul, R.: Kupamupindi, T.: Labadarios, D. (2015) Body image and weight control in South Africans 15 years or older: SANHANES-1. *BMC Public Health* 15, 992.

52. Mogre V, Mwinlenna PP Oladele J (2013) Distorted self-perceived weight status and its associated factors among civil servants in Tamale, Ghana: a cross-sectional study. *Archives of Public Health* 71, 30.

53. Mogre VA, S.: Nyaba, R. (2015) Misperception of weight status and associated factors among undergraduate students. *Obes Res Clin Pract* 9, 466-474.

54. Muhihi AJ, Njelekela MA, Mpembeni R *et al.* (2012) Obesity, overweight, and perceptions about body weight among middle-aged adults in Dar es Salaam, Tanzania. *ISRN obesity* 2012.

55. Mukeshimana MMN, Z. Z. (2014) Communities' knowledge and perceptions of type two diabetes mellitus in Rwanda: a questionnaire survey. *J Clin Nurs* 23, 541-549.

56. Muzindutsi PF, Nishimwe-Niyimbanira R Sekhampu TJ (2014) Perceived benefits and barriers to physical exercise : a comparative analysis of first year and senior students at a South African University. *African Journal for Physical Health Education, Recreation and Dance* 20, 169-181.

57. Nambaka JEK, Jane: Andanje, Mwisukha: Amusa, Lateef O: Goon, Daniel T (2011) Factors influencing participation in physical exercise by the elderly in Eldoret West District, Kenya: physical activity and health. *African Journal for Physical Health Education, Recreation and Dance* 17, 462-472.

58. Nansseu JR, Petnga SN, Atangana CP *et al.* (2019) The general public's knowledge of diabetes mellitus: A cross-sectional study in Cameroon. *Primary care diabetes* 13, 97-105.

59. Ndambo MK, Nyondo-Mipando AL Thakwalakwa C (2022) Eating behaviors, attitudes, and beliefs that contribute to overweight and obesity among women in Lilongwe City, Malawi: a qualitative study. *BMC Womens Health* 22, 216.

60. Ndejjo R, Musinguzi G, Nuwaha F *et al.* (2022) Understanding factors influencing uptake of healthy lifestyle practices among adults following a community cardiovascular disease prevention programme in Mukono and Buikwe districts in Uganda: A qualitative study. *PLoS ONE [Electronic Resource]* 17, e0263867.

61. Nizeyimana EP, JS (2006) Perceived constraints to physical activity among students at paramedical institutions in Uganda: health promotion, fitness and wellness. *African Journal for Physical Health Education, Recreation and Dance* 12, 394-402.

62. Nolan V, Sandada M Surujlal J (2011) Perceived benefits and barriers to physical exercise participation of first year university students. *African Journal for Physical Health Education, Recreation and Dance* 17, 56-69.

63. Nolan VS, J (2011) Participation in physical activity: An empirical study of undergraduate university students' perceptions. *African Journal for Physical Health Education, Recreation and Dance* 17, 70-85.

64. Odukoya OO, Odediran O, Rogers CR *et al.* (2022) Barriers and Facilitators of Fruit and Vegetable Consumption among Nigerian Adults in a Faith-Based Setting: A Pre-Intervention Qualitative Inquiry. *Asian Pacific Journal of Cancer Prevention: Apjcp* 23, 1505-1511.

65. Ojofeitimi E, Adeyeye A, Fadiora A *et al.* (2007) Awareness of obesity and its health hazard among women in a university community. *Pakistan Journal of Nutrition* 6, 502-505.

66. Okeke EI, GN: Ene-Obong, HN (2006) Body weight perception among Igbo people in the University of Nigeria, Nsukka. *Agro-Science* 5.

67. Okop KJM, F. C.: Mathole, T.: Levitt, N.: Puoane, T. (2016) Perceptions of body size, obesity threat and the willingness to lose weight among black South African adults: a qualitative study. *BMC Public Health* 16, 365.

68. Okop KJ, Levitt N Puoane T (2019) Weight underestimation and body size dissatisfaction among black African adults with obesity: Implications for health promotion. *African journal of primary health care & family medicine* 11, e1-e8.

69. Okoro EOO, B. A.: Etebu, E. N.: Sholagberu, H.: Kolo, P. M.: Chijioke, A.: Adebisi, S. A. (2014) Body size preference among Yoruba in three Nigerian communities. *Eat Weight Disord* 19, 77-88.

70. Olaoye OR & Oyetunde OO (2012) Perception of weight and weight management practices among students of a tertiary institution in south west Nigeria. *Journal of Applied Pharmaceutical Science* 2, 81.

71. Osiberu AA, Oluwasanu MM, Omobowale M *et al.* (2021) A cross-sectional study of the knowledge and screening practices of diabetes among adults in a south western Nigerian city. *J Prev Med Hyg* 62, E529-e538.

72. Oyeyemi ALA, B. O.: Oyeyemi, A. Y.: Sallis, J. F. (2011) Perceived environmental correlates of physical activity and walking in African young adults. *Am J Health Promot* 25, e10-19.

73. Peltzer K (2001) Healthy dietary practices among black South African university students. *Health SA Gesondheid* 6, 59-65.

74. Peltzer K (2002) Healthy dietary practices among rural and semi-urban Blacks in the Northern Province of South Africa. *Curationis* 25, 41-47.

75. Peltzer K (2002) Healthy dietary practices among black and white South Africans. *Ethnicity & Disease* 12, 336-341.

76. Peltzer K (2003) Body image and physical activity among black university students in South Africa. *African Journal for Physical Activity and Health Sciences* 9, 208-217.

77. Karl Peltzer K-N (2004) Nutrition knowledge among a sample of urban black and white South Africans. *South African Journal of Clinical Nutrition* 17, 24-31.

78. Peltzer K & Supa P (2006) Perceived benefits of physical exercise behaviour among black university students. *African Journal for Physical Health Education, Recreation and Dance* 12, 60-69.

79. Pengpid SP, Karl (2012) Body weight and body image among a sample of female and male South African university students. *Gender and Behaviour* 10, 4509-4522.

80. Peltzer K & Pengpid S (2015) Underestimation of weight and its associated factors in overweight and obese university students from 21 low, middle and emerging economy countries. *Obes Res Clin Pract* 9, 234-242.

81. Peltzer KP, S. (2004) Knowledge, barriers, and benefits of fruit and vegetable consumption and lay conceptions of nutrition among rural and semi-urban Black South Africans. *Psychol Rep* 94, 976-982.

82. Phetla MCS, L. (2017) Perceptions of healthcare professionals regarding their own body weight in selected public hospitals in Mpumalanga Province, South Africa. *S Afr Med J* 107, 338-341.

83. Phillips EAC, D. L.: Pisa, P. T.: Stein, A. D.: Norris, S. A. (2016) Perceptions of diet, physical activity, and obesity-related health among black daughter-mother pairs in Soweto, South Africa: a qualitative study. *BMC Public Health* 16, 750.

84. Prioreschi AW, S. V.: Cohen, E.: Reddy, A.: Said-Mohamed, R.: Twine, R.: Tollman, S. M.: Kahn, K.: Dunger, D. B.: Norris, S. A. (2017) Examining the relationships between body image, eating attitudes, BMI, and physical activity in rural and urban South African young adult females using structural equation modeling. *PLoS One* 12, e0187508.

85. Prioreschi A, Wrottesley SV Norris SA (2021) Physical Activity Levels, Food Insecurity and Dietary Behaviours in Women from Soweto, South Africa. *Journal of Community Health* 46, 156-164.

86. Puoane T, Fourie J, Shapiro M *et al.* (2005) ‘Big is beautiful’–an exploration with urban black community health workers in a South African township. *South African Journal of Clinical Nutrition* 18, 6-15.

87. Puoane T, Matwa P, Hughes G Bradley HA (2006) Socio-cultural factors influencing food consumption patterns in the black African population in an urban township in South Africa.

88. Ramautar Y, Tlou B Dlungwane TP (2021) Knowledge, attitudes and practices of hospital-based staff regarding physical activity at a private hospital in Johannesburg. *S* 63, e1-e7.

89. Rutebemberwa EK, S. K.: Gitta, S. N.: Mwaka, A. D.: Atuyambe, L. (2013) Perceptions of diabetes in rural areas of Eastern Uganda. *Curationis* 36, E1-7.

90. Shiferaw WS, Gatew A, Afessa G *et al.* (2020) Assessment of knowledge and perceptions towards diabetes mellitus and its associated factors among people in Debre Berhan town, northeast Ethiopia. *PloS one* 15, e0240850.

91. Siervo MG, P.: Nyan, O. A.: Prentice, A. M. (2006) A pilot study on body image, attractiveness and body size in Gambians living in an urban community. *Eat Weight Disord* 11, 100-109.

92. Simfukwe PVW, B.: Swart, C. (2017) Perceptions, attitudes and challenges about obesity and adopting a healthy lifestyle among health workers in Pietermaritzburg, KwaZulu-Natal province. *Afr J Prim Health Care Fam Med* 9, e1-e9.

93. Skaal LP, S. (2011) Obesity and health problems among South African healthcare workers: Do healthcare workers take care of themselves? *South African Family Practice* 53, 563-567.

94. Tuakli-Wosornu YAR, M.: Gittelsohn, J. (2014) Perceptions of physical activity, activity preferences and health among a group of adult women in urban Ghana: a pilot study. *Ghana medical journal* 48, 3-13.

95. Tumusiime D & Frantz JM (2006) Influence of previous participation in physical activity on its perceptions among tertiary institution students: physical activity and health. *African Journal for Physical Health Education, Recreation and Dance* 12, 287-297.

96. Tuoyire DAK-K, A.: Doku, D. T.: Amo-Adjei, J. (2018) Perceived ideal body size of Ghanaian women: "Not too skinny, but not too fat". *Women Health* 58, 583-597.

97. van den Berg VL, Okeyo AP, Dannhauser A Nel M (2012) Body weight, eating practices and nutritional knowledge amongst university nursing students, Eastern Cape, South Africa. *African Journal of Primary Health Care & Family Medicine* 4, 323.

98. Venter FC, Walsh CM, Slabber M Bester CJ (2009) Body size perception of African women (25-44 years) in Manguang. *Journal of Consumer Sciences* 37.

99. Walter CMDR, Rosa (2011) Socio-cultural barriers to physical activity among black isixhosa speaking professional women in the nelson mandela metropolitan municipality. *South African Journal for Research in Sport, Physical Education and Recreation* 33, 143-155.

100. Yepes M, Maurer J, Stringhini S *et al.* (2016) Ideal body size as a mediator for the gender-specific association between socioeconomic status and body mass index: evidence from an upper-middle–income country in the African region. *Health Education & Behavior* 43, 56S-63S.

101. Yiga P, Ogwok P, Achieng J *et al.* (2021) Determinants of dietary and physical activity behaviours among women of reproductive age in urban Uganda, a qualitative study. *Public Health Nutrition* 24, 3624-3636.
